# Structural insights into the action mechanisms of artificial electron acceptors in photosystem II

**DOI:** 10.1016/j.jbc.2023.104839

**Published:** 2023-05-19

**Authors:** Shinji Kamada, Yoshiki Nakajima, Jian-Ren Shen

**Affiliations:** 1 Faculty of Science, Okayama University, Okayama, Japan; 2 Research Institute for Interdisciplinary Science and Graduate School of Natural Science and Technology, Okayama University, Okayama, Japan

**Keywords:** Photosystem II, photosynthesis, electron transfer, structural biology, crystal structure, electron acceptor

## Abstract

Photosystem II (PSII) utilizes light energy to split water, and the electrons extracted from water are transferred to Q_B_, a plastoquinone molecule bound to the D1 subunit of PSII. Many artificial electron acceptors (AEAs) with molecular structures similar to that of plastoquinone can accept electrons from PSII. However, the molecular mechanism by which AEAs act on PSII is unclear. Here, we solved the crystal structure of PSII treated with three different AEAs, 2,5-dibromo-1,4-benzoquinone, 2,6-dichloro-1,4-benzoquinone, and 2-phenyl-1,4-benzoquinone, at 1.95 to 2.10 Å resolution. Our results show that all AEAs substitute for Q_B_ and are bound to the Q_B_-binding site (Q_B_ site) to receive electrons, but their binding strengths are different, resulting in differences in their efficiencies to accept electrons. The acceptor 2-phenyl-1,4-benzoquinone binds most weakly to the Q_B_ site and showed the highest oxygen-evolving activity, implying a reverse relationship between the binding strength and oxygen-evolving activity. In addition, a novel quinone-binding site, designated the Q_D_ site, was discovered, which is located in the vicinity of Q_B_ site and close to Q_C_ site, a binding site reported previously. This Q_D_ site is expected to play a role as a channel or a storage site for quinones to be transported to the Q_B_ site. These results provide the structural basis for elucidating the actions of AEAs and exchange mechanism of Q_B_ in PSII and also provide information for the design of more efficient electron acceptors.

Photosystem II (PSII) exists in thylakoid membranes of oxygenic photosynthetic organisms and performs light-induced electron transfer reactions coupled with water splitting to produce electrons, protons, and oxygen molecules (1, 2, 3). The chemical energy converted and oxygen produced by PSII are essential for life on earth. Cyanobacterial PSII consists of 17 transmembrane subunits, 3 or 4 peripheral proteins (4, 5, 6), and a number of pigments and cofactors, with a total molecular weight of around 350 kDa for a monomer. The structure of a PSII dimer has been analyzed by X-ray crystallography at 1.9 Å resolution from the thermophilic cyanobacterium *Thermostichus (Thermosynechococcus) vulcanus*, which revealed the arrangement of all protein subunits and the precise location of all cofactors including chlorophylls (Chls), carotenoids, and an oxygen-evolving manganese–calcium cluster, providing a basis for elucidating the function of PSII. Recently, single-particle analysis using cryoelectron microscopy revealed the PSII structure in solution at 1.93 to 1.95 Å resolution, which is closer to the *in vivo* state of PSII and was found to be essentially identical to PSII in its crystalline state with some minor differences (6, 7).

When PSII receives light energy from the sun, the Chls referred to as P680 in the reaction center are excited (2), which leads to the stable charge separation. Electrons are ejected from P680, which becomes P680^+^. P680^+^ pulls electrons from a catalytic center called a manganese–calcium cluster (Mn_4_CaO_5_ cluster) *via* a redox-active, nearby tyrosine residue D1-Tyr161 (2). The Mn_4_CaO_5_ cluster functions as a catalyst for the splitting of water molecules into oxygen, protons, and electrons, and the high potential of P680^+^ acts as a driving force for the stepwise water-splitting reaction at the Mn_4_CaO_5_ cluster (2, 3, 4, 5). On the other hand, the electrons released from P680 are transferred to pheophytin and then to two plastoquinone (PQ) molecules, Q_A_ and Q_B_, bound to the D2 and D1 subunits, respectively (8, 9, 10) (Fig. 1, *A* and *B*). After accepting two electrons, Q_B_ is protonated with two protons transferred from the cytosol (stroma) through a hydrogen-bonding network formed by surrounding amino acids, bicarbonate (HCO_3_^−^), and water molecules, resulting in the formation of PQH_2_ (4, 5, 11, 12, 13), which leaves PSII. The empty Q_B_-binding site (Q_B_ site) is refilled with a new PQ from the PQ pool within the thylakoid membrane; in this way PQ is reduced one after another (14). The PQH_2_ released into the membrane passes electrons to the cytochrome *b*_6_*f* complex (15, 16), which are eventually used for the reduction of carbon dioxide into sugars. The majority of herbicides inhibiting photosynthetic electron transport bind to the Q_B_ site (17, 18, 19, 20, 21, 22, 23).

**Figure 1:**
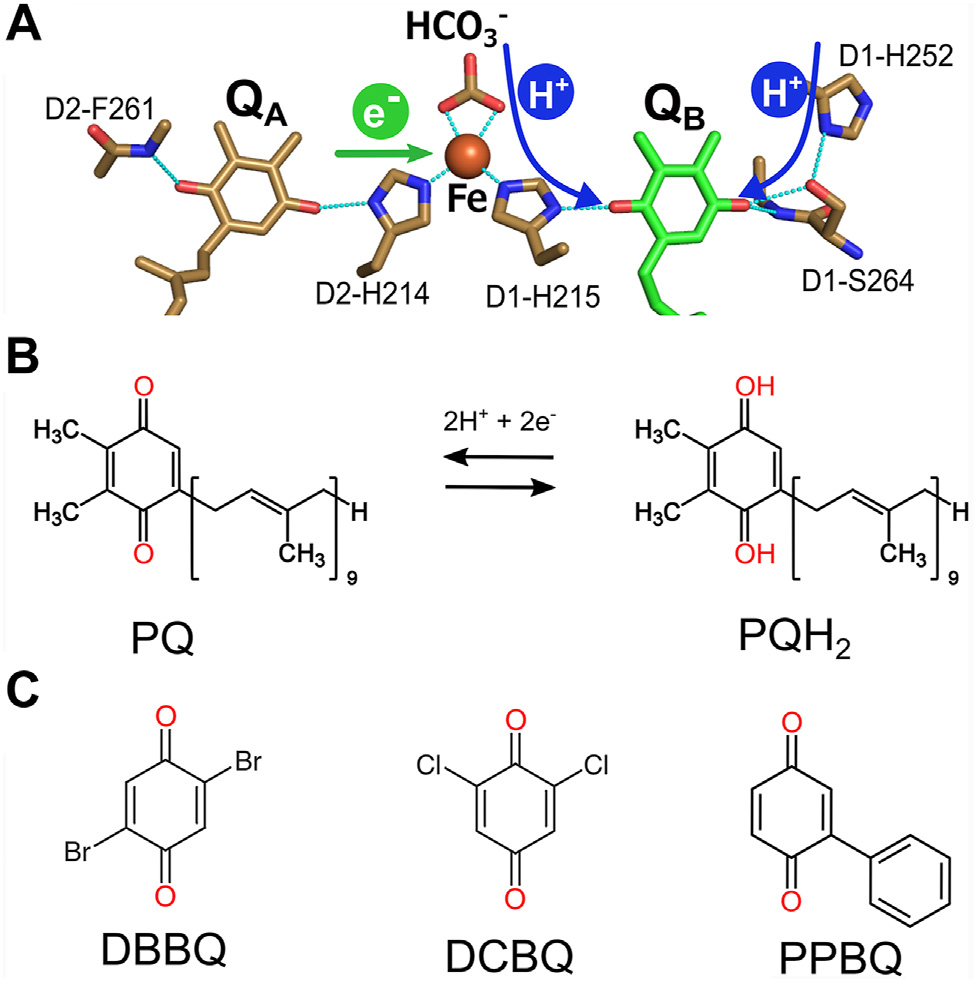
**Structures of the acceptor side of PSII and artificial electron acceptors used in this study.***A*, electron flow at the PSII acceptor side. Color code: iron, *orange*; carbon, *gold* and *green*; oxygen, *red*; nitrogen, *blue*. Coordination to the iron atom and hydrogen bonding are indicated by cyan dotted lines. *B*, native plastoquinone (PQ) acceptor in PSII and its protonation reaction. *C*, structures of artificial electron acceptors used in this study. DBBQ, 2,5-dibromo-1,4-benzoquinone; DCBQ, 2,6-dichloro-1,4-benzoquinone; PPBQ, 2-phenyl-1,4-benzoquinone.

The detailed mechanism of action of Q_B_ has been analyzed and discussed based on the protein structure and molecular dynamics analysis (2, 3, 4, 5, 6, 7, 24, 25, 26). Furthermore, a slight movement of the Q_B_ molecule during and after reduction has been directly observed by pump-probe time-resolved crystallography (27, 28, 29). However, it remained unclear how Q_B_ receives electrons at the Q_B_ site and by what mechanism it is released and rebound to the binding site after it receives two electrons. Here we focused on the action mechanism of artificial electron acceptors (AEAs) in the Q_B_ site. The molecular structure of AEAs is similar to that of the head group of Q_B_ (Fig. 1*C*), and, like natural Q_B,_ they can accept electrons to support the redox reaction of PSII (30, 31, 32, 33, 34). However, whether AEAs actually bind to PSII and how they function have not been captured at the molecular level.

In this study, PSII crystals were incubated in a solution containing three different AEAs, 2,5-dibromo-1,4-benzoquinone (DBBQ), 2,6-dichloro-1,4-benzoquinone (DCBQ), and 2-phenyl-1,4-benzoquinone (PPBQ), respectively (Fig. 1*C*), for several hours, and then subjected to X-ray crystallography. Structural analysis showed that all AEAs bind to the Q_B_ site and there was a reverse correlation between the binding strength and oxygen-evolving activity. In addition, a novel quinone-binding site was found at a position located slightly away from the Q_B_ site as well as the previously found Q_C_ site (26). These results provide important insights into the mechanism of AEAs and Q_B_ exchange in PSII as well as a hint for the design of electron acceptors in constructing artificial photosynthetic systems.

## Results

### Binding states of AEAs whose positions are detected by anomalous difference maps

**Figure 2:**
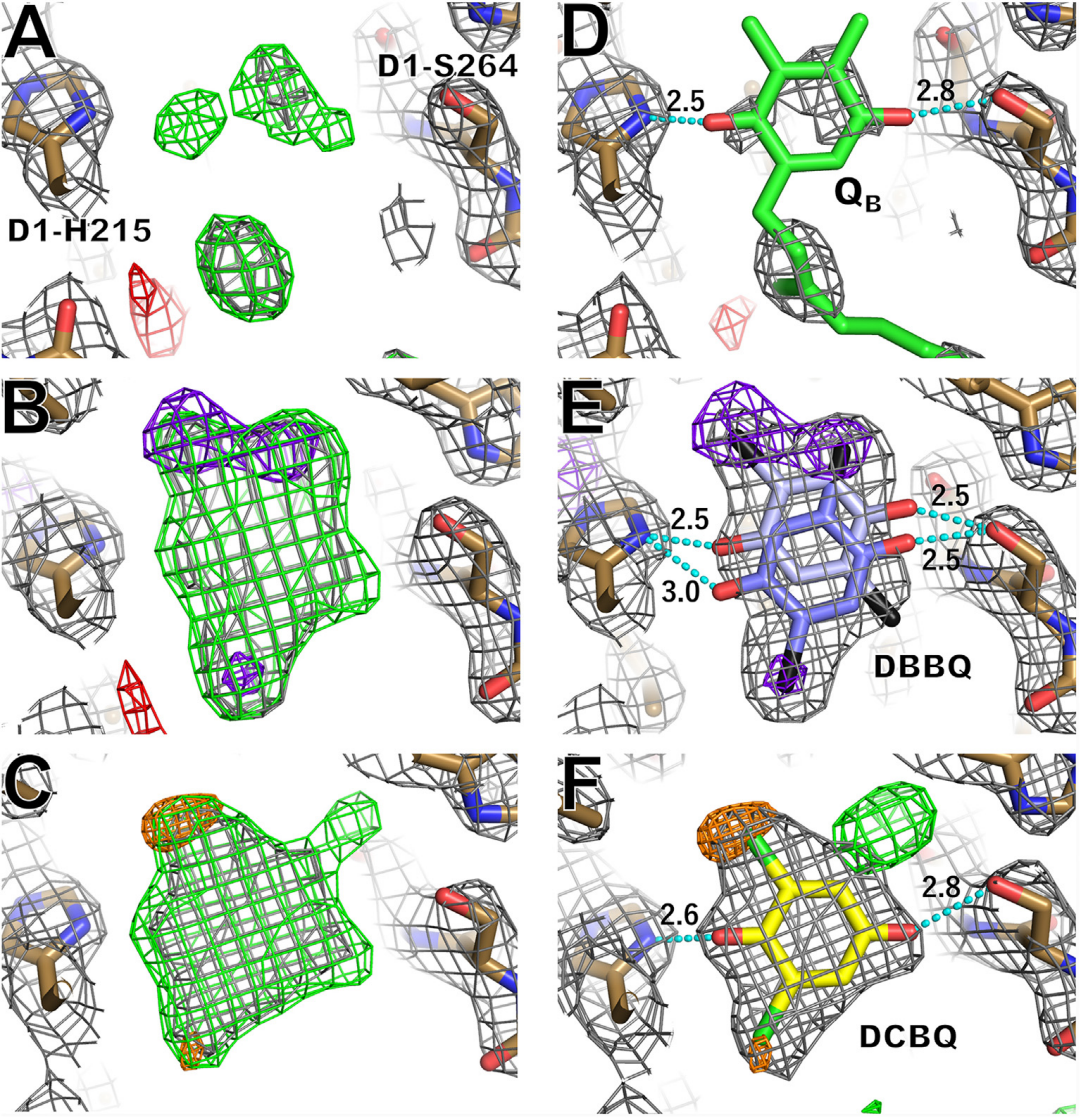
**Electron density maps with the Q**_**B**_**molecule-omitted (left column) or the structure assigned as Q**_**B**_**, DBBQ, or DCBQ molecules (right column).***A*, control condition. *B*, 10 mM DBBQ-treated condition. *C*, 10 mM DCBQ-treated condition. *D*, Q_B_ assigned map in the control condition. Q_B_ is colored in *green*. *E*, DBBQ assigned map in the 10 mM DBBQ-treated condition. DBBQ-A conformer is colored in *blue*, and DBBQ-B conformer in *light blue*, and the Br atoms are colored in *black* in both conformations. *F*, DCBQ assigned map in the 10 mM DCBQ-treated condition. DCBQ is colored in *yellow* and the Cl atoms in *light green*. The 2Fo-Fc maps (*gray*) are contoured at 1.0 σ, and Fo-Fc maps (positive, *green*; negative, *red*) are contoured at 3.5 σ. The anomalous signals of Br atoms (*purple*) are contoured at 4.5 σ, and anomalous signals of Cl atoms (*orange*) are contoured at 3.5 σ. Dotted lines (cyan) indicate the hydrogen bond, and the numerals are hydrogen-bonding distances (Å). The maps and structures shown are for the A-monomer of the PSII dimer.

X-ray diffraction images were collected from PSII crystals treated with either DBBQ or DCBQ, and the images were successfully analyzed at 2.10-Å resolution for the 10 mM DBBQ-treated condition and 2.15-Å resolution for the 10 mM DCBQ-treated condition, respectively (Table S1). The 2Fo-Fc electron density map and positive Fo-Fc map with the Q_B_ molecule omitted showed shapes different from that of the Q_B_ molecule at the Q_B_-binding site (Q_B_ site) in both AEA-treated conditions (Fig. 2, *A*–*C*). These maps matched with DBBQ and DCBQ, respectively, in the DBBQ- and DCBQ-treated conditions (Fig. 2, *D*–*F*), indicating the binding of DBBQ and DCBQ directly to the Q_B_ site in place of Q_B_. To examine the exact binding position of DBBQ and DCBQ to the Q_B_ site, Br atoms in DBBQ and Cl atoms in DCBQ were detected by X-ray anomalous dispersion, which is a specific and sensitive method to detect heavy atoms in crystals. In the DBBQ-treated condition, three anomalous signals derived from bromide atoms were found in the Q_B_ site with the diffraction data collected at 0.9 Å wavelength, which is close to the Br absorption edge (Fig. 2*B*), indicating that DBBQ adopts two conformations in the Q_B_ site. The two conformations that best match with the electron density map and the location of the anomalous signals are shown in Figure 2*E*, in which the A conformer has a 0.55 occupancy and the B conformer has a 0.45 occupancy, respectively (Fig. S1), and the average B-factor is 96.7 Å^2^. This structure is registered at the Protein Data Bank (PDB) with a PDB code of 8GN0.

On the other hand, two heavy atom–derived anomalous signals were found at the Q_B_ site in the anomalous difference map of 10 mM DCBQ-treated PSII crystals collected at 1.8 Å wavelength (Fig. 2*C*). As shown in Figure 2*F*, the electron density map and the anomalous signals fit well with a single DCBQ molecule with 1.0 occupancy, and the B-factor is 84.8 Å^2^. This structure is registered at PDB with a code of 8GN1. However, a positive Fo-Fc map remains in the upper right corner of the DCBQ, and this feature is more pronounced on the B monomer side of the PSII dimer (Fig. S2). Partial flipping of D1-His215 could explain this Fo-Fc map (see Discussion section).

Both DBBQ and DCBQ molecules assigned are within hydrogen-bonding distances of D1-His215 and D1-Ser264, similar to that observed for the natural Q_B_ molecule (Fig. 2).

### Interactions between PPBQ and the Q_B_ site

**Figure 3:**
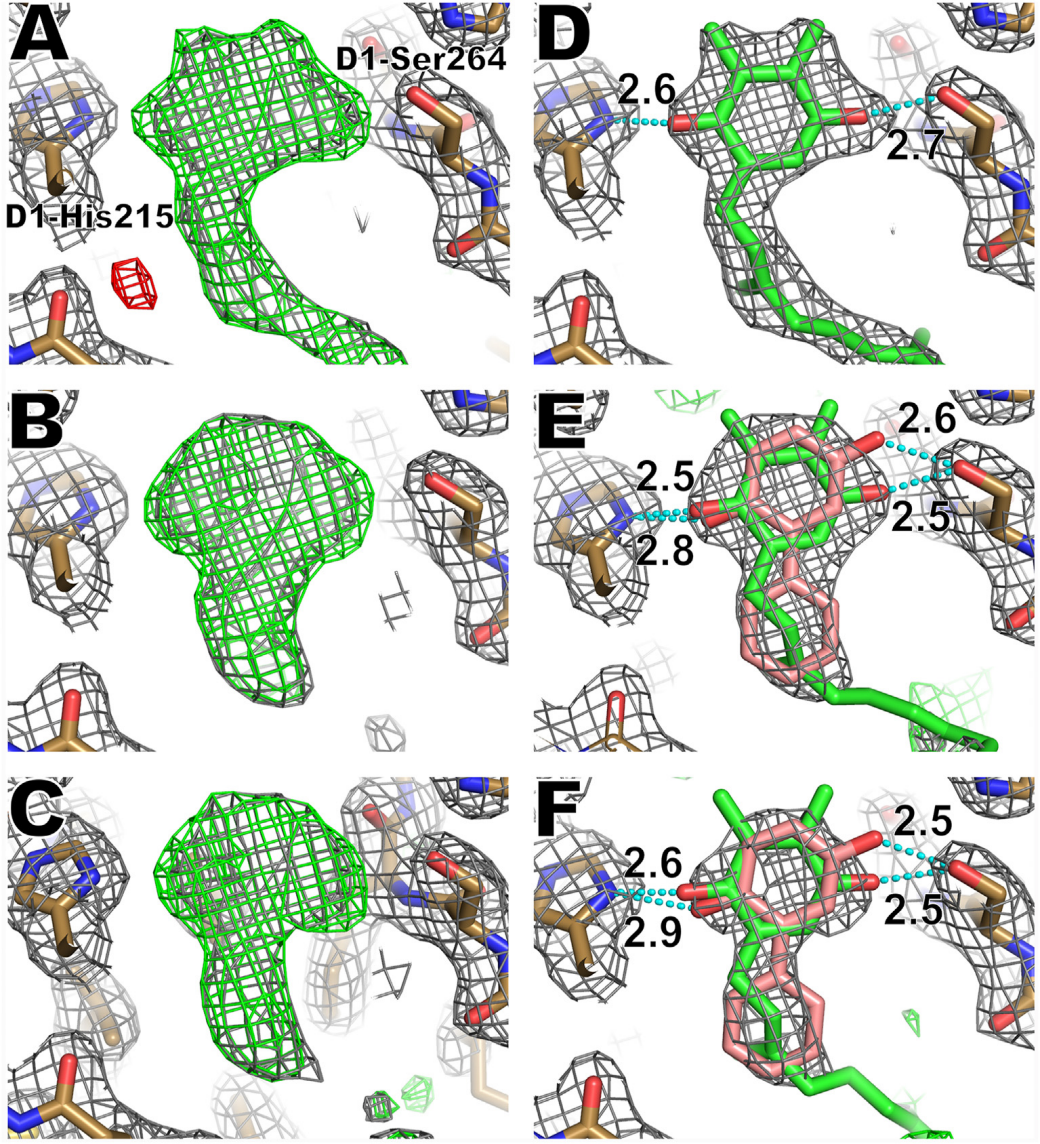
**Electron density maps in the Q**_**B**_**site and comparisons between the control and PPBQ-treated conditions.** (*Left column*) Q_B_-omitted maps. (*Right column*) Density maps after placement of the Q_B_ molecule or Q_B_ plus PPBQ. *A* and *D*, control condition. *B* and *E*, 1 mM PPBQ-treated condition. *C* and *F*, 10 mM PPBQ-treated condition. In *E* and *F*, the occupancies were set at 0.2 for PPBQ and 0.8 for Q_B_, respectively. The colors and σ levels are the same as those in Figure 2, except that the PPBQ molecule is colored in *pink*.

**Figure 4:**
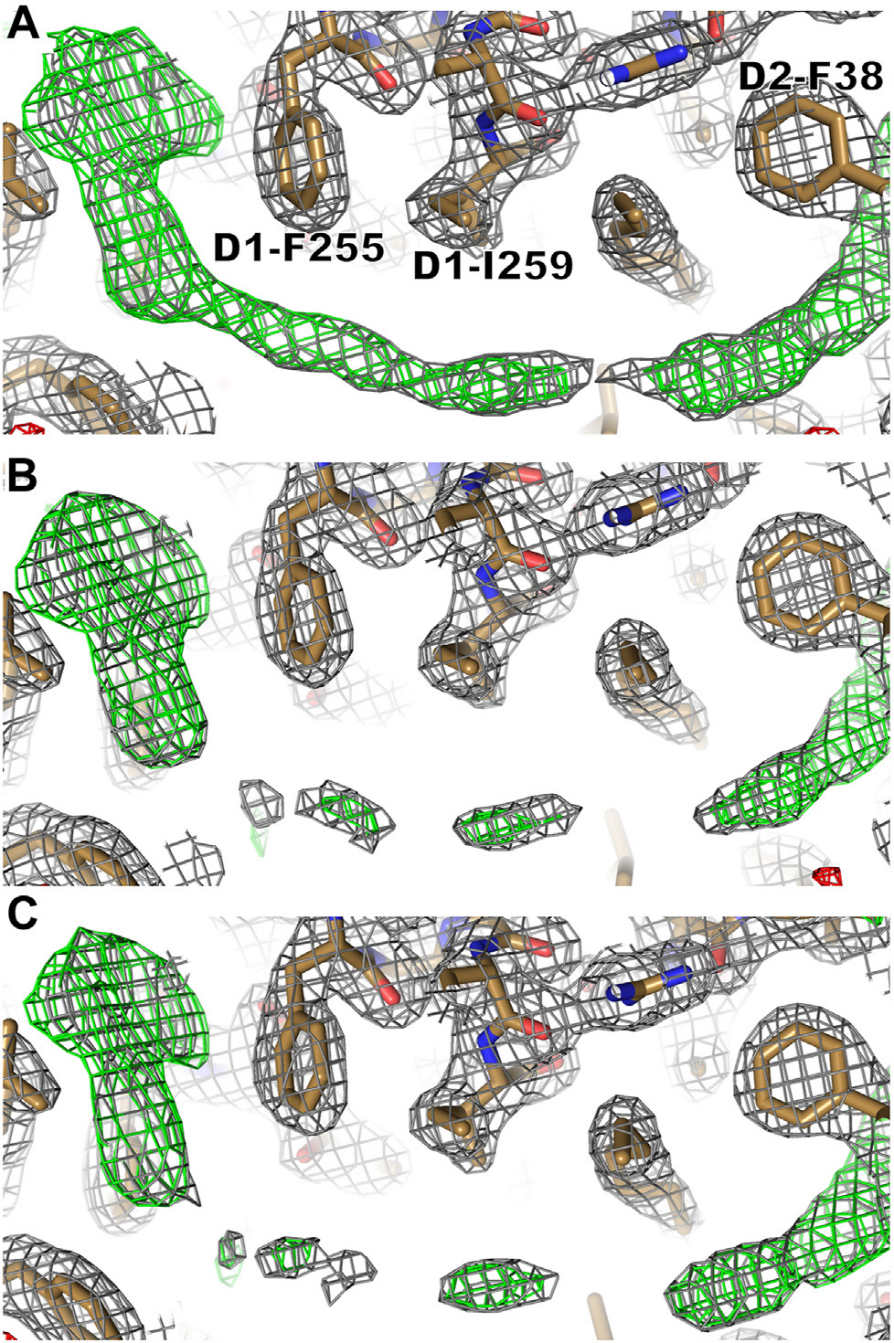
**Side view of the Q**_**B**_**and PPBQ-omitted maps in the whole Q**_**B**_**region.***A*, control. *B*, 1 mM PPBQ-treated condition. *C*, 10 mM PPBQ-treated condition. The colors and σ levels are the same as those in Figure 2.

Since PPBQ does not contain heavy atoms that show anomalous signals, the shape of the electron density map is carefully compared with that of the control condition to reveal the possible binding of PPBQ to the Q_B_ site. Initially, the electron density map obtained from crystals with PPBQ added by the same cryoprotectant as for DBBQ and DCBQ failed to show apparent binding of PPBQ compared with the control (Figs. 2*A* and S3). This result does not agree with several previous studies, including electron paramagnetic resonance, thermoluminescence, and fluorescence measurements, which suggests the binding of PPBQ to the Q_B_ site (35, 36, 37, 38). This discrepancy was found to be due to the presence of dimethyl sulfoxide (DMSO), an organic solvent, which has been used as a cryoprotectant reagent at a high concentration. This makes PPBQ to be dissolved in DMSO and hence difficult to occupy the Q_B_ site. After the replacement of DMSO by glycerol as a cryoprotectant, the data obtained from PPBQ-treated crystals showed a clear PPBQ-like electron density map instead of Q_B_ in the Q_B_ site (Fig. 3). The Q_B_ omit map is shown in Figure 3, *A* and *B*, and C for the control, 1 mM PPBQ-treated, and 10 mM PPBQ-treated conditions, respectively. In the control condition, the map clearly showed the shape of the PQ head (Fig. 3*A*), whereas in the PPBQ-treated conditions, the map changed to a round shape and the map corresponding to the tail of the Q_B_ molecule was largely obscured or no longer visible (Fig. 3, *B* and *C*). Figure 4 shows the map corresponding to the tail of Q_B_ at different angles, in which a large area of the Q_B_ tail is obscured in the PPBQ-treated conditions compared with the control condition. Next, maps assigned with the Q_B_ molecule (PQ) were compared (Fig. S4, *A*–*F*). The electron density map observed in the control condition was in good agreement with the shape of the Q_B_ molecule. On the other hand, in the PPBQ-added conditions, the maps corresponding to the two methyl groups in the head region and tail of Q_B_ were unclear or broken (Fig. S4, *B* and *C*), so were the tail regions of the molecule from the side view (Fig. S4, *D*–*F*). These results suggest that the Q_B_ molecule has been replaced by the PPBQ molecule in the PPBQ-treated conditions.

To quantitatively compare the fit of the Q_B_ molecule with the map in each condition, the average B-factors of the Q_B_ molecule were compared between the control and PPBQ-treated conditions, assuming Q_B_ occupies its position in all the conditions. The average B-factor of the Q_B_ molecule was found to be higher under the PPBQ-treated condition than that under the control condition (Table 1). A plausible explanation for this is that, in the PPBQ-treated condition, part of the Q_B_ molecule leaves the Q_B_ site and is replaced by PPBQ, resulting in the increasing of the Q_B_ B-factor. To test this possibility, the density map was recalculated with a lower occupancy of the Q_B_ molecule in the control and PPBQ-treated conditions. Fig. S4, *G*–*I* shows the refined map with the Q_B_ occupancy reduced to 0.6. In the control condition, the Fo-Fc map shows a small signal throughout the Q_B_ molecule, consistent with a binding of Q_B_ in full occupancy (Fig. S4*G*). On the other hand, in the PPBQ-treated conditions, one relatively large Fo-Fc positive area was observed near the neck of the Q_B_ head (Fig. S4, *H* and *I*). Since the crystals were prepared under the same condition except for the addition of PPBQ, a plausible interpretation of this large positive area is the presence of PPBQ. Assuming PPBQ is present at this position, this large positive area corresponds to the second benzene ring of the PPBQ (Fig. 1*C*).

**Table 1:** Average B-factors (Å^2^) of the Q_B_ molecule in PPBQ-treated conditions

Condition	Entire	Head
Control	75.1	63.9
1 mM PPBQ treated	77.6	84.9 (+33%)
10 mM PPBQ treated	84.5	95.7 (+50%)

The head refers to the C1 to C6 atoms that make up the benzene ring, the two oxygen atoms, CH3 that makes up the two horns, and the C7 atom that connects the head to the tail.

To verify the binding of PPBQ to the Q_B_ site, PPBQ molecules were assigned to the map for each condition and refined (Fig. S5, *A*–*C*). Since PPBQ was not added to the crystal in the control condition, two positive Fo-Fc map areas were observed in the region corresponding to the two methyl groups of the Q_B_ head region (Fig. S5*A*) and a negative Fo-Fc area was observed at the location of the second benzene ring. Furthermore, a large and long positive Fo-Fc map is visible in the area corresponding to the tail of Q_B_. On the other hand, in both PPBQ-added conditions, no such strong Fo-Fc areas were observed in the positions observed in the control condition (Fig. S5, *B* and *C*). These results indicate that it is more reasonable to assign PPBQ to the Q_B_ site than to assign only Q_B_ under the PPBQ-treated conditions.

However, as shown in Fig. S5, *B* and *C*, a small Fo-Fc positive area was still observed even after refinement with the assignment of PPBQ. Assuming that this small Fo-Fc area was due to residual Q_B_ molecule, the PPBQ and Q_B_ multiconformers were assigned with different occupancies and refined. As a result, the placement of PPBQ and Q_B_ as multiconformers matched well with the map features (Fig. 3, *E* and *F*). The ratio of multiconformers was determined based on the Fo-Fc map, and it was found that PPBQ binds to the Q_B_ site by replacing up to 60% of Q_B_ (Fig. S5, *D*–*N*). Structures whose occupancy ratio is 0.2 for PPBQ and 0.8 for Q_B_, where the B-factor is not too high (Table S2), are shown in Figure 3, *E* and *F*, and the structure of 10 mM PPBQ-treated condition is registered at PDB with a code of 8GN2. The binding positions of PPBQ revealed that the hydrogen bond distances between the two carbonyl groups of PPBQ and D1-His215, D1-Ser264 are not significantly different from those of the native Q_B_.

### Anomalous signals outside of the Q_B_ site

**Figure 5:**
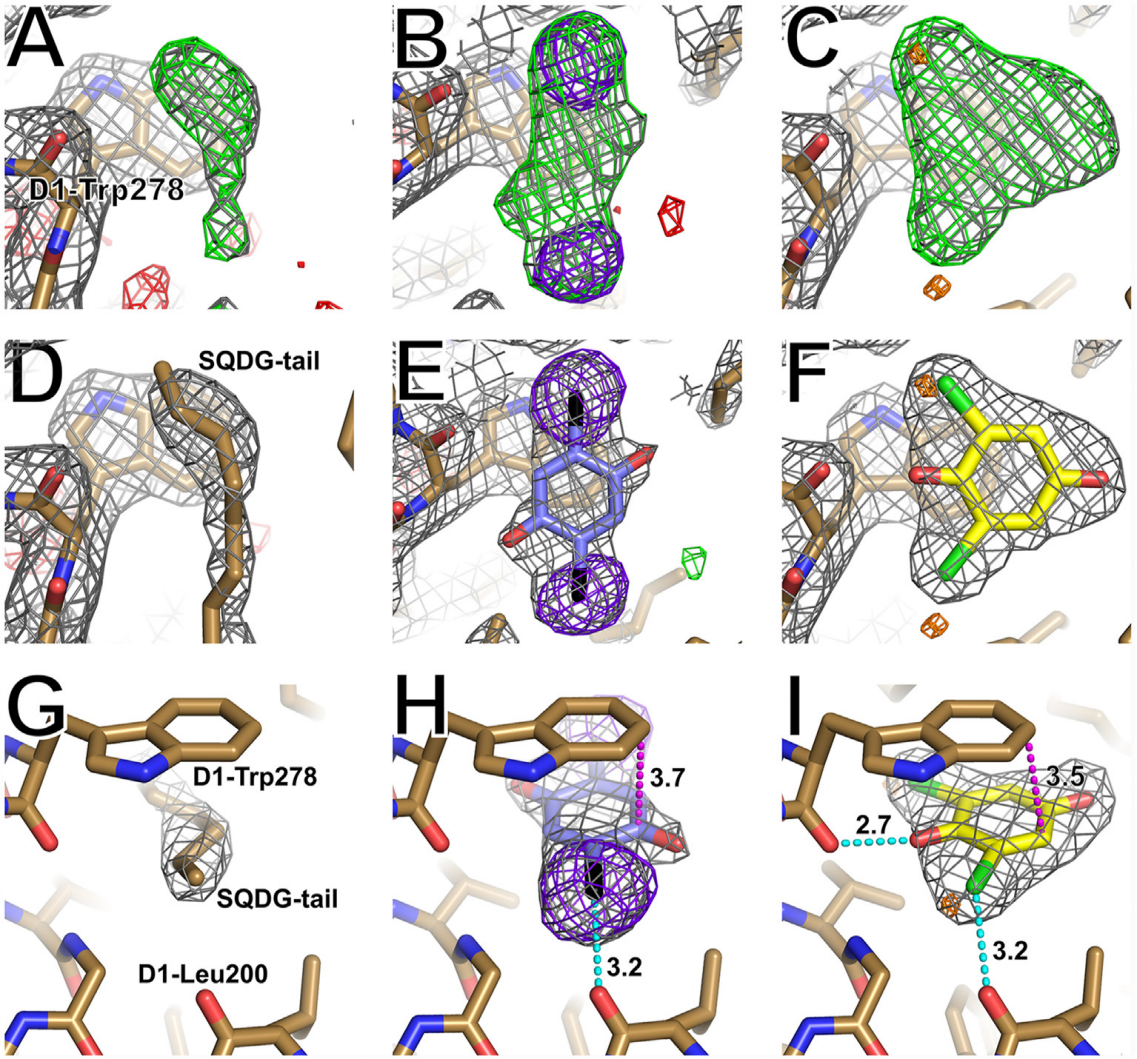
**Electron density maps at the Q**_**D**_**site in each condition.***A*, *D*, and *G*, control. *B*, *E*, and *H*, 10 mM DBBQ-treated condition. *C*, *F*, and *I*, 10 mM DCBQ-treated condition. Cyan dotted lines indicate hydrogen bond between the artificial electron acceptors and amino acid residues in the proposed Q_D_ site, and the numerals are bond distances (Å). The magenta dotted lines and numerals represent the π–π stacking interactions between artificial electron acceptors and D1-Trp278. The colors and σ levels are the same as those in Figure 2.

**Figure 6:**
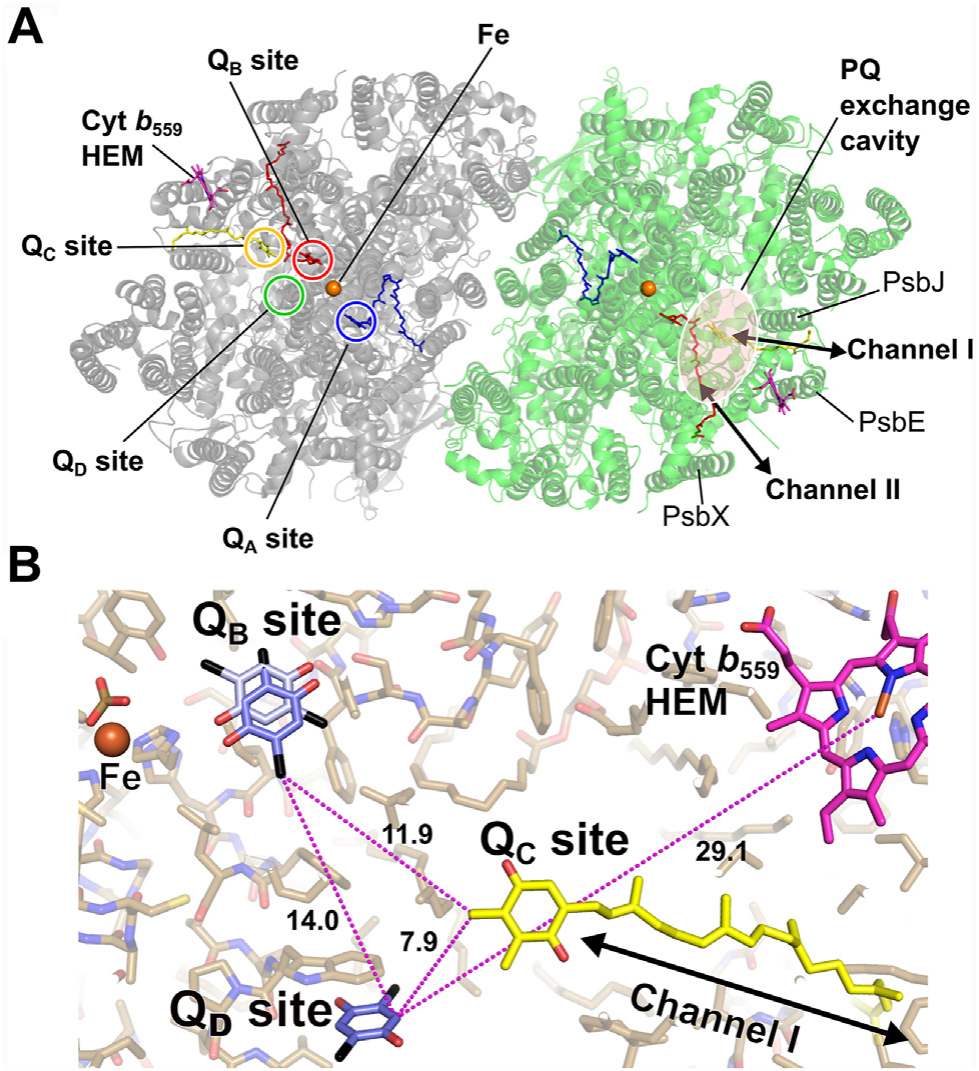
**All of the quinone-binding sites in PSII.***A*, the locations of quinone-binding sites, the quinone exchange channels, and cytochrome *b*_559_ (Cyt *b*_559_). *B*, the distances between Q_B_, Q_C_, and Q_D_ sites. The model of the DBBQ-treated structure was superimposed with the model of the previous study (4V62) (26), and only the Qc molecules (*yellow*) are shown from the 4V62 model. The distances (Å) between the quinone-binding sites are shown in dotted lines in magenta.

Two new, strong heavy-atom anomalous signals were found at a location other than the Q_B_ site in the 0.9 Å X-ray diffraction data of DBBQ-treated crystal (Fig. 5*B*). This position is occupied by the tail of an SQDG molecule, which is surrounded by D1-Trp278 and D1-Leu200 in control PSII (Fig. 5, *A*, *D* and *G*), and is separated from the Q_B_ site by around 15 Å. In the DBBQ-treated PSII, the 2Fo-Fc map at this position showed a DBBQ-like shape different from that observed in the control condition (Fig. 5, *A* and *B*). Based on this 2Fo-Fc map and the anomalous signals, a DBBQ molecule was assigned to this map. The distance between the two anomalous signals is consistent with the distance between the two Br atoms in the DBBQ molecule (Fig. 5*E*). This novel quinone-binding site is called the Q_D_-binding site (Q_D_ site) in this article to distinguish from the Q_C_ site reported previously (26). The distance between Q_D_ and the head of Q_B_ is around 14 Å and that between Q_D_ and Q_C_ is around 8 Å (Fig. 6). A Q_D_ site has been proposed previously based on two phase reduction of cytochrome *b*_*559*_ (39, 40). The Q_D_ site designated in this study may be either the same or different from the previously reported Q_D_ site, although the Q_D_ site identified here has a distance of around 29 Å to cytochrome *b*_*559*_ (Fig. 6*B*).

Similar results were obtained from the DCBQ-treated crystal (Fig. 5, *C*, *F* and I). Smaller anomalous signals were observed for the DCBQ-treated crystal, as these signals were collected with a wavelength of 1.8 Å, which is much shorter than the K-edge absorption of Cl atom at 4.4 Å. The shape of the 2Fo-Fc map in the Q_D_ site well matches with that of the DCBQ molecule, thus they are assigned as the DCBQ molecule in the DCBQ-treated PSII. Because an occupancy of 1.0 for either the DBBQ or DCBQ-treated PSII exhibited a negative Fo-Fc map, their respective occupancies were investigated for the Q_D_ site. The occupancies were determined by the Fo-Fc map to be 0.65 for DBBQ and 0.9 for DCBQ (Fig. S6).

### Oxygen-evolving activities with different AEAs

To examine the activities of PSII with the different AEAs used in this study, the oxygen-evolving activity in the presence of DBBQ, DCBQ, or PPBQ was measured (Table 2). PPBQ showed an activity of 3100 μmol O_2_/mg Chl/h, which is the highest among the three AEAs. When PPBQ was used as a reference, DBBQ was 17% less active and DCBQ was 43% less active.

**Table 2:** Comparison of oxygen-evolving activities between different AEAs

Accepter	O_2_-evolving activity (μmol O_2_/mg Chl/h)	% PPBQ activity
PPBQ	3100 ± 60	100
DBBQ	2600 ± 50	83
DCBQ	1800 ± 60	57

Oxygen evolution of the purified PSII dimers was measured three times independently and averaged with their standard deviations listed.

## Discussion

### Binding of AEAs to the Q_B_-binding site and the oxygen-evolving activity

All of the three AEAs examined in this study appeared to bind to the Q_B_-binding site (Q_B_ site) in place of Q_B_. The binding of DBBQ is well supported by the X-ray anomalous dispersion data measured at 0.9 Å wavelength, which gives rise to the largest anomalous signal of the Br atoms derived from the DBBQ molecule. Two sites were found to bind the DBBQ molecule; these two sites are in close proximity, so they are modeled as multiconformations of a single molecule (Fig. 2, *B* and *E*). The shape of the 2Fo-Fc map matched well with that of the two DBBQ molecules. The distances of DBBQ to the nearby residues do not change much in the two different conformations. Similar results were obtained for DCBQ (Fig. 2, *C* and *F*), which bind to a single site in place of Q_B_. Compared with the anomalous signal of the Br atom in DBBQ, the anomalous signal of the Cl atom in DCBQ was smaller. This is because the K absorption edge of the Cl atom is 4.4 Å, whereas the dataset was collected at 1.8 Å wavelength under the DCBQ-added condition. As the Cl ions in the vicinity of the manganese cluster have been identified by the similar approach in previous studies (4, 41, 42), this method is effective in identifying the Cl atoms. The 2Fo-Fc map also matched with the DCBQ molecule, thereby supporting the binding of DCBQ at the Q_B_ site.

The binding of PPBQ to the Q_B_ site is less straightforward, as it does not contain a heavy atom that can be used to identify its position by anomalous diffraction. Nevertheless, as described in the results section, a detailed comparison of the electron density maps and B-factors of the PPBQ treated condition and the control showed that PPBQ can bind to the Q_B_ site (Figs. 3 and 4; Table 1, Figs. S4 and S5; and Table S2).

**Figure 7:**
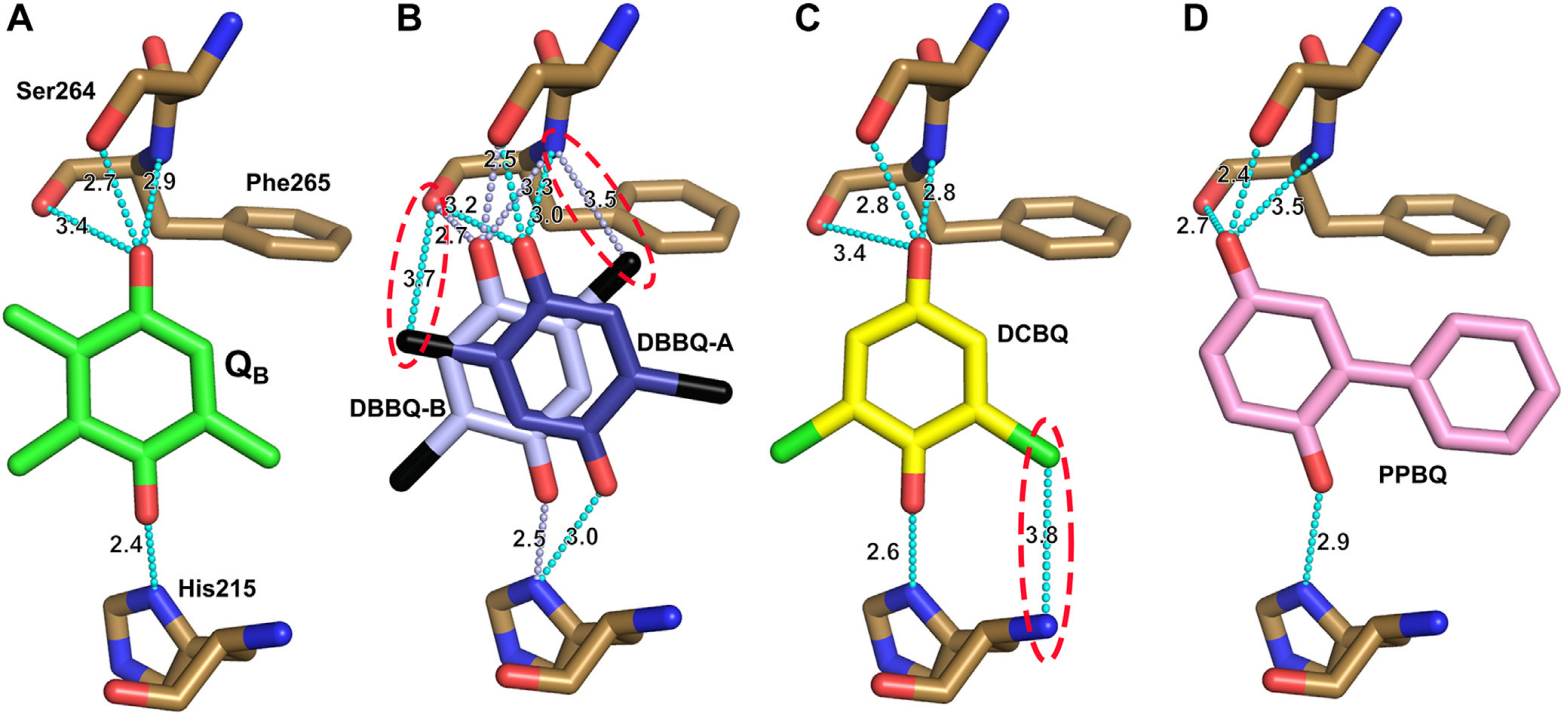
**Interactions of Q**_**B**_**and artificial electron acceptors with the nearby amino acid residues.** (*A*) Q_B_; (*B*) DBBQ; (*C*) DCBQ; (*D*) PPBQ. *Red* dashed circles indicate “halogen bonds.” Hydrogen and halogen bonds are colored in cyan, but only those of DBBQ B-conformer are colored in *light blue*.

The above results indicate that all three AEAs can bind to the Q_B_ site and receive electrons in place of Q_B_. This is consistent with several previous studies using thermoluminescence, fluorescence, and electron paramagnetic resonance measurements that demonstrated the binding and action of AEAs, including PPBQ, on the Q_B_ site (35, 36, 37, 38). In addition, these AEAs are within hydrogen bonding distances to D1-His215, Ser264, and Phe265, which are similar as those seen in the natural Q_B_ (Fig. 7). This may suggest a similar protonation mechanism of AEAs as that of Q_B_ (11, 12, 13, 43). These results suggest that AEAs support the electron transport of PSII by binding to the Q_B_ site in place of Q_B_, receiving electrons, and replacing themselves one after another.

Differences are found in the oxygen-evolving activities among the three AEAs employed, with PPBQ giving the highest activity, whereas DCBQ has the lowest activity (Table 2). This result is consistent with previous studies comparing the activity of different AEAs (30, 31). We should point out that, although the activities are measured in the presence of potassium ferricyanide in the present study, the quinone-type AEAs alone without the presence of potassium ferricyanide should give a similar trend, as the quinone-type AEAs alone support the majority of the activity, and supplement of potassium ferricyanide only slightly enhanced the activity (44).

The differences in the oxygen-evolving activities supported by different AEAs can be explained by the differences in the binding strength of AEAs to the Q_B_ site. Comparisons between the electron density maps of the crystals treated with 10 mM AEAs showed that the shape of the DCBQ molecule appeared most clear, whereas the shape of the PPBQ molecule was not visible clearly, and DBBQ was in between (Figs. 2, *B* and *C* and S3). A simple comparison of the binding strength of AEAs to the Q_B_ site in terms of the map strength shows that the order is DCBQ≥DBBQ > PPBQ, which is inversely proportional to the order of the oxygen-evolving activity that each AEA supported. This difference can be explained based on the attachment of the bromide or chloride ions. As shown in Figure 7, the two conformations of DBBQ form an additional “halogen bond” between their bromide ions and the main-chain nitrogen or oxygen atom of D1-Phe265, and DCBQ forms an additional halogen bond between its chloride ion and the main-chain nitrogen atom of D1-His215. However, PPQB does not form any additional bond to the nearby residues due to the absence of bond-formable atoms, and it has two phenol rings compared with one ring of other AEAs or Q_B_. Thus, the binding strength of PPBQ to the Q_B_ site is the lowest among the three AEAs. As both DBBQ and DCBQ form additional halogen bond with the nearby residues, they can fully replace Q_B_ in the Q_B_ site. The difference of the binding strength between DCBQ and DBBQ may be due to the multiconformations of DBBQ found in the Q_B_ site, which makes the binding of each molecule of DBBQ weaker. These observations suggest that the AEAs must have a low binding strength for the Q_B_ site in order for them to fully support the oxygen-evolving activity. This weaker binding strength may contribute to the rapid detachment of AEAs from the Q_B_ site after receiving electrons, hence facilitating the binding of next AEAs. These results suggest that designing an AEA that has a lower binding strength may further enhance the activity of PSII.

In the DCBQ-treated condition, a positive Fo-Fc map remains in the upper right corner of the DCBQ, and this feature is more pronounced on the B monomer side of PSII dimer (Fig. S2). A partial inversion of D1-His215 can explain this Fo-Fc map, but DCBQ and D1-His215 cannot exist simultaneously because they are located too close so that they will collide. The most optimal occupancy ratio was determined based on the Fo-Fc map to be 0.6 for DCBQ and 0.4 for D1-His215 in the B monomer side. On the other hand, in the A monomer side, the 2Fo-Fc map corresponding to the flipped D1-His215 was not visible and hence the flipped D1-His215 could not be modeled. Therefore, in the A monomer, DCBQ is thought to occupy most of the Q_B_ sites.

### AEA-binding sites other than the Q_B_ site

In addition to the Q_B_ site, there are many other locations on PSII where anomalous signals are observed. Most of them arise from cofactors such as nonheme iron between Q_A_ and Q_B_, heme iron at cytochrome *b*_*559*_ and cytochrome *c*_*550*_, and manganese clusters found in previous studies (4, 5). However, two new anomalous signals that could not be interpreted as PSII cofactors were found in the map of the DBBQ-treated crystal collected at 0.9 Å wavelength X-ray (Fig. 5*B*). In native PSII, there is no heavy atom at this position that emits anomalous signals, and the tail of SQDG is assigned in this region in the previous studies (4, 5) as well as in the control condition in this study (Fig. 5, *D* and *G*). Therefore, these anomalous signals are most likely derived from the Br atoms of DBBQ. The shape of the 2Fo-Fc density map at this position is consistent with the binding of DBBQ (Fig. 5, *B*, *E* and H). Thus, a DBBQ molecule was assigned in this position; concomitant with the occupation by DBBQ, the tail of the SQDG molecule shifted away from its original position. A similar result was obtained from the crystal treated with DCBQ (Fig. 5, *C*, *F* and I). These AEAs are in a position to have π-π stacking interactions with D1-Trp278. The Br atom of DBBQ is at a hydrogen bond distance of 3.18 Å to the carbonyl group of D1-Leu200, and the ketone of DCBQ is at a hydrogen bond distance of 2.69 Å to the carbonyl group of D1-Trp278. These intermolecular interactions are thought to stabilize the AEAs at this position. This site is termed the Q_D_ site, which is around 8 Å away from the third quinone-binding site (Q_C_ site) found in a previous structural analysis (26) (Fig. 6). The Q_C_ site is located in channel I (26), one of the proposed PQ exchange pathways, and the Q_D_ site is also close to channel I. Previous studies have suggested that the Q_C_ site is a region for temporarily retaining quinone destined for the Q_B_ site (8, 26, 45, 46, 47). However, the Q_C_ site exists in a fairly hydrophobic environment, and there is no polar contact or π-π stacking that would stabilize the headgroup orientation of the quinone. In contrast, in the Q_D_ site, the head group of the quinone interacts with surrounding amino acid residues and therefore may be more stable. Thus, the Q_D_ site may be more appropriate to temporarily hold part of the quinone that goes to the Q_B_ site or serve as a channel for Q_B_ to be transported to its binding site, and the Q_C_ may be a quinone that has temporarily derived from the Q_D_ site. In support of this hypothesis, all structures obtained by high-resolution X-ray crystallography and cryo-EM, including this study, do not contain Q_C_ (4, 5, 6, 7). It should be noted, however, that even at high concentrations (10 mM AEAs) the occupancies of the Q_D_ site are below 1.0, indicating a low occupancy of the Q_D_ site *in vivo*. In either case, both Q_C_ and Q_D_ sites are rather close with each other, suggesting that this area may be the site for Q_B_ to pass through or storage.

The existence of the Q_D_ site was predicted based on a kinetic study of cytochrome b_559_ reduction (39, 40). According to this study, the Q_D_ site is located in the vicinity of cytochrome *b*_559_ and affects its redox reaction. However, the Q_D_ site we found is around 30 Å away from cytochrome *b*_559_ and is separated from it by a hydrophobic PQ exchange cavity. Therefore, it is possible that the Q_D_ site we discovered in the structure is different from the Q_D_ site predicted based on the spectroscopic studies. However, we cannot exclude the possibility that the two Q_D_ sites represent the same one.

Like AEAs, herbicides such as 3-(3, 4-dichlorophenyl)-1, 1-dimethylurea bind to the Q_B_ site and inhibit photosynthesis (17, 18, 19, 20, 21, 22, 23). A previous study showed that herbicides bind to another site in addition to the Q_B_ site (48). It was thought previously that this was the Q_C_ site to which the natural quinone binds, but our results suggest that it may also be the Q_D_ site that we found here. To confirm this hypothesis, it will be necessary to analyze the PSII crystal structure at high resolution under inhibitor-treated conditions. In a previous study, crystal structure analysis under terbutryn-added condition was performed at a resolution of 3.2 Å (21). However, this resolution is insufficient to reveal the exact structure of the binding site and weakly bound molecules. From the method used in this study, PSII crystal structures with AEAs fully bound to the Q_B_ and Q_D_ sites were analyzed. This method may be applicable to analyze the crystal structures of herbicide-bound PSII at a high resolution.

## Experimental procedures

### Cell culture and purification

Thermophilic cyanobacterium *Thermostichus (Thermosynechococcus) vulcanus* was cultured in a liquid medium at 50 °C with bubbling of air containing 3% (v/v) CO_2_ under LED light in a plant growth chamber (BIOTRON LH-410PF-SP) as described (44, 49). For a large-scale culture, 40 L of cells was grown for 7 days, and the light intensity was increased gradually from 50 to 130 μmol photons m^−2^ s^−1^. The cultured cells were harvested and disrupted by lysozyme treatment and a freeze-thawing method (44, 49). Extraction and purification of highly active, dimeric PSII were performed according to a previously reported procedure (44, 49), and it was finally stored in a buffer containing 5% (w/v) glycerol, 20 mM Mes-NaOH (pH 6.0), 20 mM NaCl, 3 mM CaCl_2_. All procedures for the preparation were performed under dim green light.

### Crystallization

The recrystallization method was used to improve the crystal quality of the PSII dimer. In the first step, the PSII core complexes were crystallized for 12 to 24 h at 5 °C in a 1.5-ml tube with the batch method, and microcrystals obtained were collected and resolubilized by a buffer containing 20 mM Mes-NaOH (pH 6.0), 20 mM NaCl, 10 mM CaCl_2_. Finally, the PSII dimer sample obtained was crystallized with the same procedure as that reported previously (4, 49, 50). All procedures for the preparation were performed under dim green light.

### Dehydration and cryoprotection

The PSII crystals obtained were subjected to the treatment of dehydration and cryoprotection as follows. The crystals were first transferred into a 100 μl buffer solution containing 7% polyethylene glycol (PEG) 1450 in addition to the crystallization buffer. After incubation at 12 °C for 15 min, 100 μl of a buffer containing 0.2% higher concentration of PEG 1450 and new 1.8% DMSO and 0.7% PEG 5000 MME was added. After 15 min incubation, half of the buffer volume was replaced with a new buffer that contained the same increment of PEGs and DMSO. This procedure was repeated every 15 min until the concentrations of DMSO and total PEGs in the final buffer reached 25% and 20%, respectively. For the structural analysis of PSII crystals treated with AEAs, DBBQ, DCBQ, and PPBQ were added to the buffer solutions, respectively. The initial buffer solution did not contain AEAs, and the concentration was increased stepwise in the same way as the increase in the concentration of PEG 5000 and DMSO, so that the final buffer solutions contained 1 mM or 10 mM of AEAs, respectively. The crystals were then dehydrated by evaporation against air at a humidity of 75 to 90% in the final buffer for 1 h in an incubator at 12 °C, flash-frozen in a nitrogen gas stream, and stored in liquid nitrogen. All cryoprotectant replacement and cryocooling procedures were carried out under dim green light.

For the PPBQ conditions, dehydration and cryoprotection treatment were also performed using a buffer containing glycerol instead of DMSO. The crystals were first transferred into a 100 μl buffer solution containing 7% PEG 1450 and 2% DMSO in addition to the crystallization buffer. After incubation at 12 °C for 15 min, 100 μl of a buffer containing 0.2% higher concentration of PEG 1450 and new 1.6% glycerol and 0.7% PEG 5000 MME were added. After 15 min incubation, half of the buffer volume was replaced with a new buffer containing the same increment of PEGs and glycerol. This procedure was repeated every 15 min until the concentrations of glycerol and total PEGs in the final buffer reached to 23% and 20%, respectively. The other procedures are the same as described above.

### Data collection and structural analysis

X-ray diffraction experiments were performed at beamline BL41XU of SPring-8, Japan, at 100 K. Most of the PSII is in the S_1_ state because the crystals were made in the dark, and X-rays were given in full darkness. Diffraction datasets were collected at a wavelength of 1.0 Å from untreated, DCBQ-treated, or PPBQ-treated PSII crystals with an oscillation angle of 0.2° over 180°, resulting in 900 images. The datasets collected were processed, integrated, and scaled using X-ray Detector Software (51). The initial phase up to 4-Å resolution was obtained by molecular replacement with the program Molrep (52) of the CCP4 suite (53) using the structure of native PSII (3WU2) as the search model. The structures of PSII crystals were refined with Refmac5 (54) of the CCP4 suite and Phenix (55, 56). Model building was performed with COOT (57, 58), and figures were drawn using PyMOL (http://www.pymol.org).

X-ray anomalous dispersion is a specific and sensitive method for detecting heavy atoms in crystals (59, 60, 61). To identify the positions of bromide ions in DBBQ and chloride ions in DCBQ, diffraction datasets were collected from the DBBQ-treated and DCBQ-treated PSII crystals at a wavelength of 0.9 Å and 1.8 Å, respectively, with an oscillation angle of 0.2° over 360°, resulting in 1800 images. The wavelength of 0.9 Å is close to the K-edge of bromide (0.92 Å, 13.47 keV), and the dataset collected at this wavelength is used to refine the structure of DBBQ-treated PSII as well as locate the bromide atoms. On the other hand, the K-absorption edge of the chloride ion is 4.4 Å, whereas the longest wavelength available at SPring-8 is around 1.8 Å, so this wavelength is used to locate the chloride ions in DCBQ-PSII. These datasets were processed with X-ray Detector Software and refined as described above. The anomalous difference Fourier maps were calculated with fast Fourier transform in the CCP4 program suite. Resolution and refinement statistics are shown in Table S1.

### Oxygen evolution measurements

Oxygen evolution of thylakoid membranes and PSII dimers was measured with a Clark-type oxygen electrode (Hansatech Instruments) under continuous, saturating illumination at 30 °C in the storage buffer at a Chl concentration of 10 μg of Chl ml^−1^. In addition to 1 mM potassium ferricyanide, 0.4 mM PPBQ, DBBQ, or DCBQ was added as an electron acceptor in the measurements. Ferricyanide was added to maximize oxygen evolution, but AEA activities are largely similar without the addition of ferricyanide (44). Measurements were taken three times, and the mean and standard deviation are shown in Table 2.

## Data availability

Atomic coordinates for the reported structures have been deposited in the Protein Data Bank under accession codes of 8GN0, 8GN1, and 8GN2 for DCBQ, DBBQ, and PPBQ-bound PSII, respectively. All other data are contained in this paper.

## Supporting information

This article contains supporting information.

## Conflict of interest

The authors declare that they have no conflicts of interest with the contents of this article.
