# Supplementary material for: Structural insights into the action mechanisms of artificial electron acceptors in photosystem II
Source: J Biol Chem. 2023 May 19;299(7):104839. doi: 10.1016/j.jbc.2023.104839 (PMC10300377; doi:10.1016/j.jbc.2023.104839)
Supplement: Supporting Figures S1–S6 and Tables S1 and S2 [file mmc1.pdf]

Supporting Information for

Structural insights into the action mechanisms of artificial electron acceptors in photosystem II

Shinji Kamada[1], Yoshiki Nakajima[2,*], Shen Jian-Ren[2,*]

[1]Faculty of Science, Okayama University, Okayama 700-8530, Japan

[2]Research Institute for Interdisciplinary Science and Graduate School of Natural Science and Technology, Okayama University, Okayama 700-8530, Japan

*Corresponding authors

Yoshiki Nakajima, E-mail: yoshi-n@okayama-u.ac.jp

Jian-Ren Shen, E-mail: shen@cc.okayama-u.ac.jp

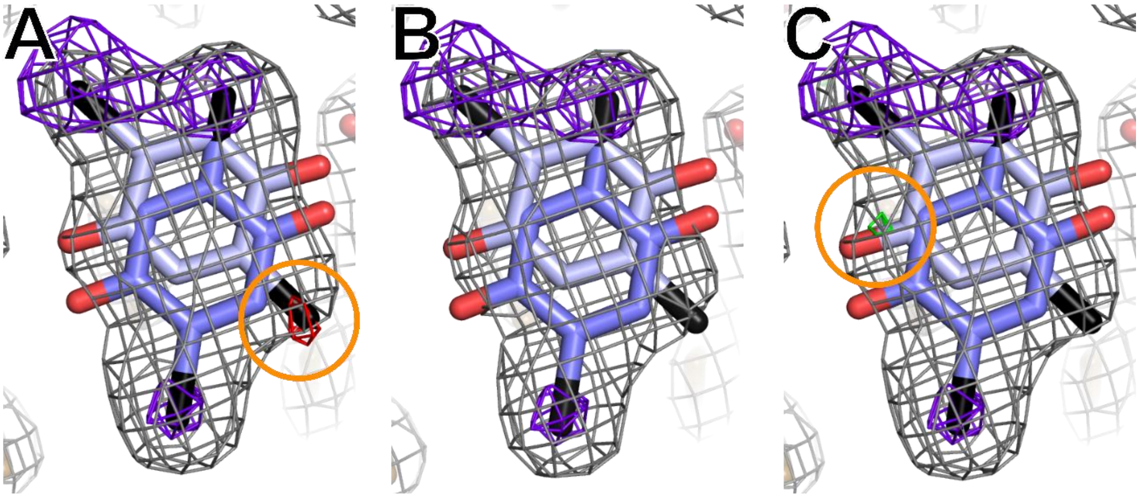

Supplementary Fig. 1. Electron density maps in the Q_B site supplemented with DDBQ, and the assignment of DBBQ with different ratios of two conformations. (A) The occupancy ratio is assigned at 0.50 for A-conformer and 0.50 for B-conformer. **(B)** The occupancy ratio is assigned at 0.55 for A-conformer and 0.45 for B-conformer. **(C)** The occupancy ratio is assigned at 0.6 for A-conformer and 0.4 for B-conformer. The 2Fo-Fc map (grey) is contoured at 1.0 σ level, and Fo-Fc map (positive map: green and negative map: red) is contoured at 3.5 σ level. Anomalous signals of Br atoms (purple) are contoured at 4.5 σ level.

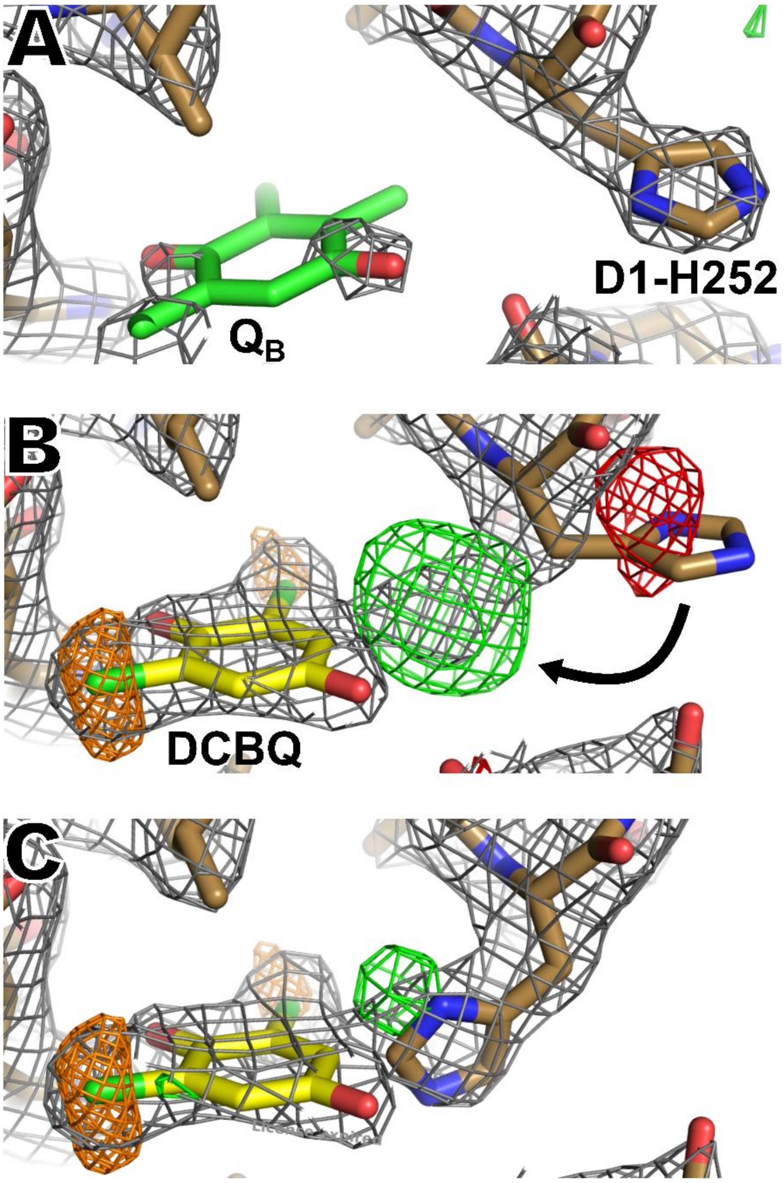

Supplementary Fig. 2. The flip of D1-His252 in the B-monomer of DCBQ-treated PSII. (A) Electron density map near Q_B and D1-His252 in the control condition. **(B)** Density map in the 10 mM DCBQ treated-condition, with D1-His252 assigned to the same conformation as the control condition. **(C)** Density map with D1-His252 flipped to resolve the positive electron density feature near DCBQ in the 10 mM DCBQ-treated condition. The optimal occupancy is 0.4 for the His252 sidechain and 0.6 for DCBQ. Anomalous signals of Cl atoms (orange) are contoured at 4.5 σ. The other colors and σ levels are the same as in Fig. S1.

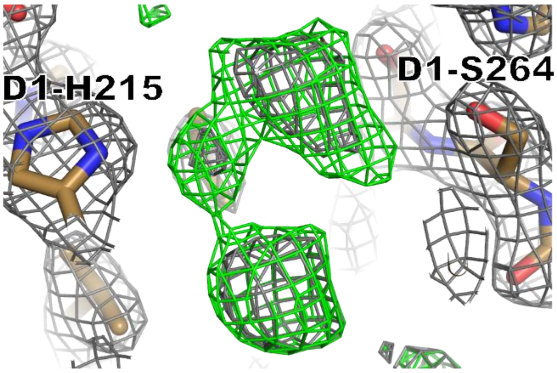

Supplementary Fig. 3. Electron density map obtained from crystals treated with 10 mM PPBQ and frozen using cryoprotectant containing DMSO. The colors and σ levels are the same as those in Fig. S1.

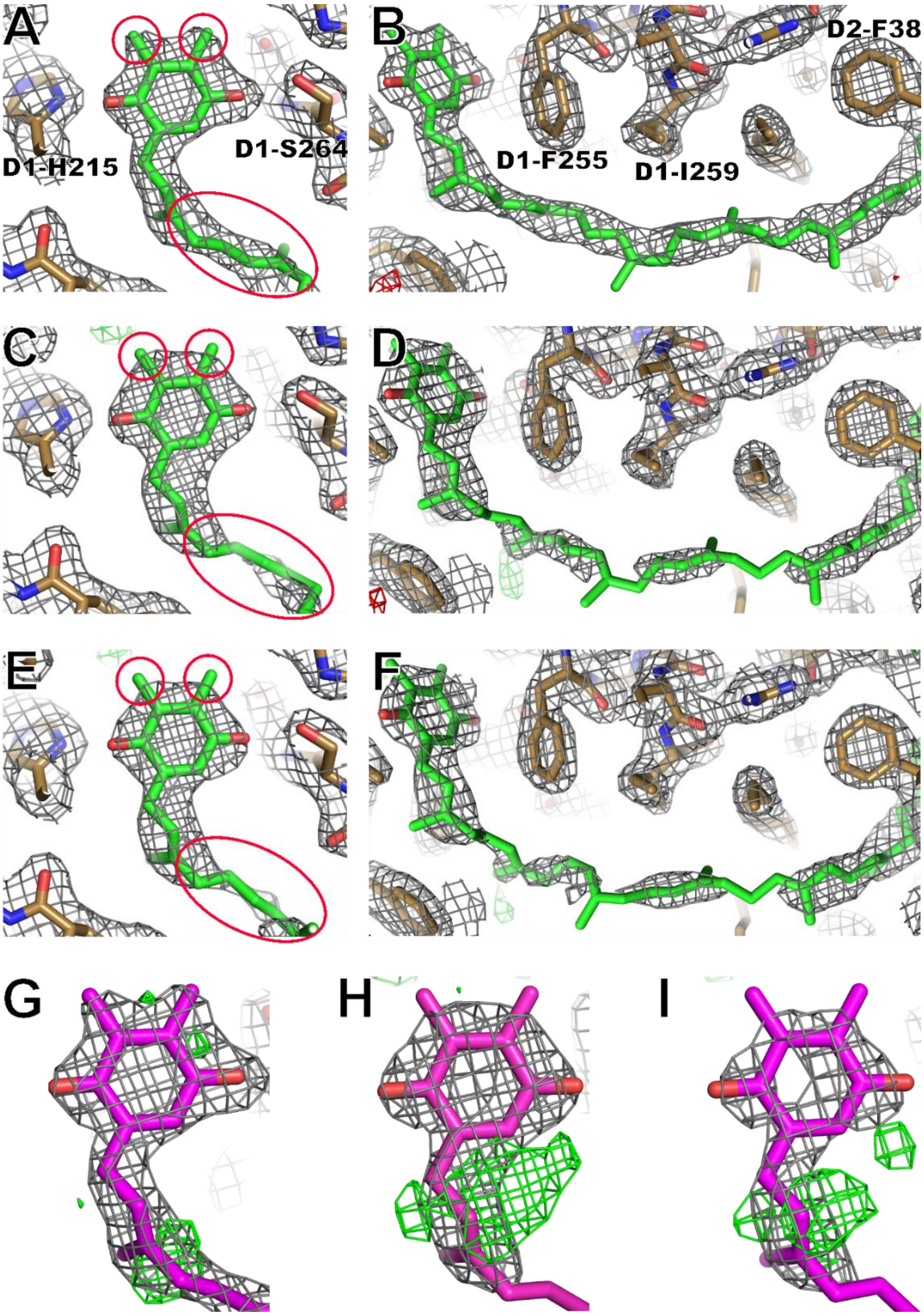

Supplementary Fig. 4. Comparison of electron density maps of the Q_B site with Q_B molecule assigned in all conditions. The Q_B molecule assigned with 1.0 occupancy in control condition **(A and B)**, in 1 mM PPBQ-treated condition (**C and D**), and in 10 mM PPBQ-treated condition (**E and F**). The Q_B site with the Q_B occupancy reduced to 0.6 in control condition **(G)**, in 1 mM PPBQ-treated condition **(H)**, and in 10 mM PPBQ-treated condition **(I)**. The colors and σ levels are the same as in Fig. S1.

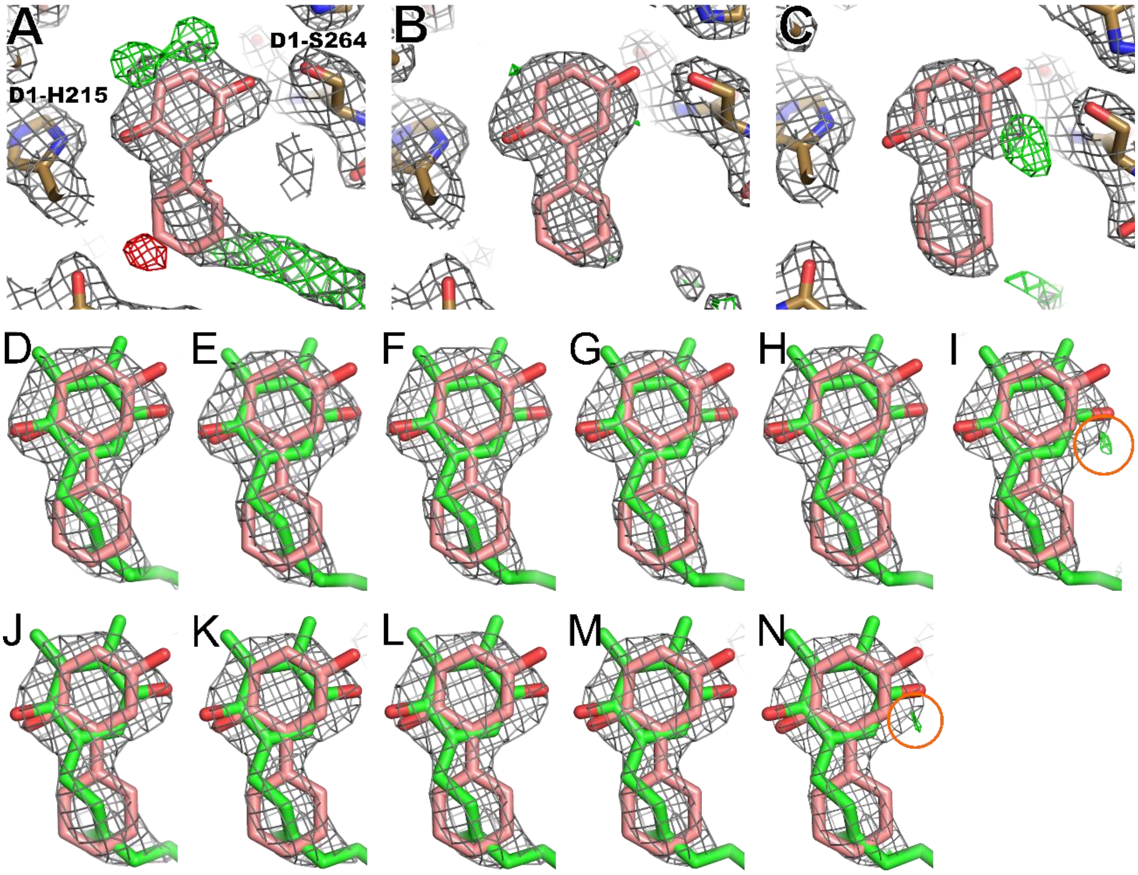

Supplementary Fig. 5. Electron density maps of the Q_B site with PPBQ assigned in all conditions, and comparison of maps when PPBQ and Q_B molecules are assigned with different occupancies. Density maps when PPBQ molecule is assigned with 1.0 occupancy in control condition **(A)**, in 1 mM PPBQ-treated condition **(B)**, and in 10 mM PPBQ-treated condition **(C)**. **(D-N)**: Density maps when PPBQ and Q_B molecules are assigned with different occupancies. (Upper low) 1 mM PPBQ-treated condition. (Lower low) 10 mM PPBQ-treated condition. **(D and J)**: The occupancy ratio is 0.1 for PPBQ and 0.9 for Q_B. **(E and K)**: The occupancy ratio is 0.2 for PPBQ and 0.8 for Q_B. **(F and L)**: The occupancy ratio is 0.3 for PPBQ and 0.7 for Q_B. **(G and M)**: The occupancy ratio is 0.4 for PPBQ and 0.6 for Q_B. **(H and N)**: The occupancy ratio is 0.5 for PPBQ and 0.5 for Q_B. **(I)**: The occupancy ratio is 0.6 for PPBQ and 0.4 for Q_B. The colors and σ levels are the same as those in Fig. S1.

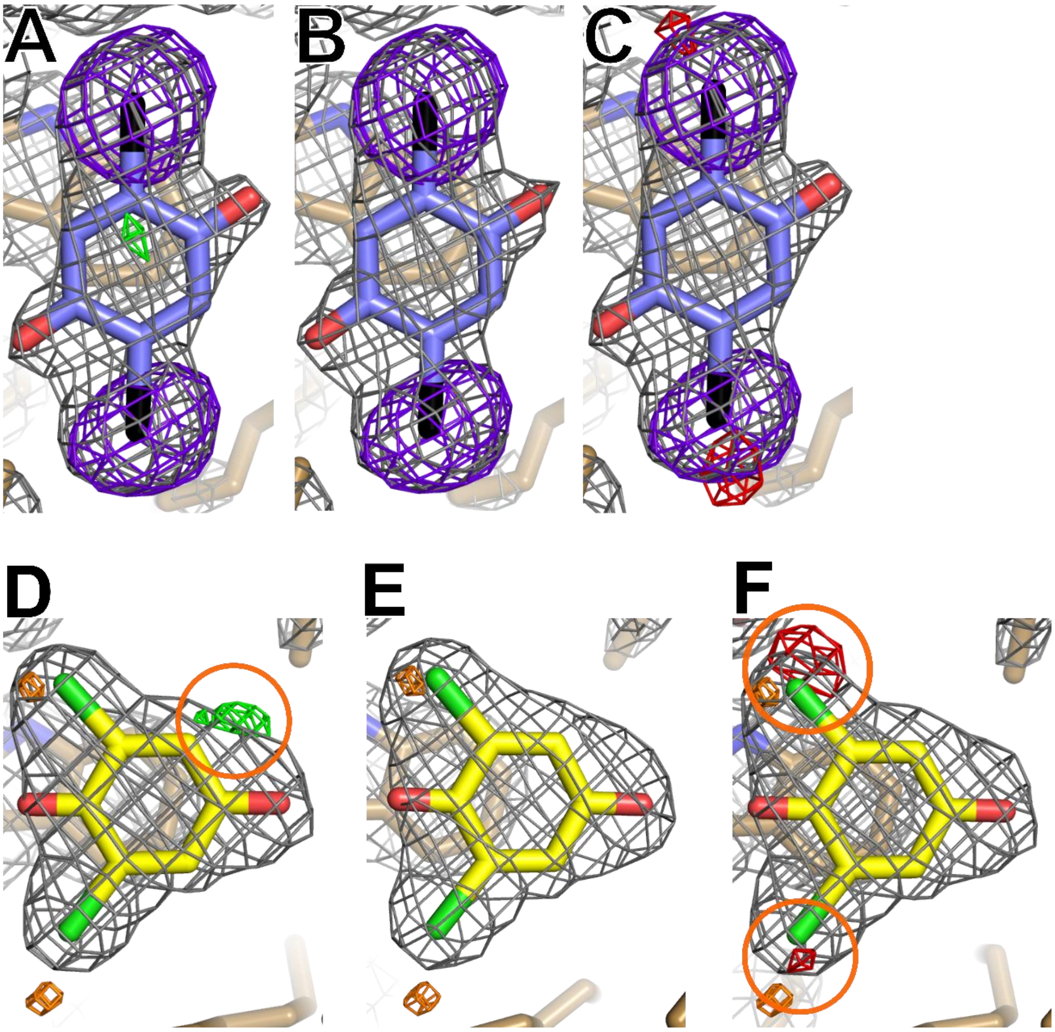

Supplementary Fig. 6. The Q_D site with DBBQ or DCBQ assigned with different occupancies in the DBBQ or DCBQ-treated PSII. The occupancy of DBBQ is set to 0.60 **(A)**, 0.65 **(B)** and 0.70 **(C)**, in DBBQ-treated condition. The occupancy of DCBQ is set to 0.8 **(D)**, 0.9 **(E)**, and 1.0 **(F)**, in DCBQ-treated condition. The colors and σ levels are the same as in Fig. S1.

Table S1. X-ray data collection and refinement statistics of X-ray crystallography (A), and X-ray anomalous dispersion (B).

A

	Control	10 mM DBBQ (PDB code: 8GN0)	10 mM DCBQ (PDB code: 8GN1)	10 mM PPBQ (DMSO)
**Data collection statistics**				
Wavelength / Å	1.00	0.90	1.00	1.00
Space group	$P2_12_12_1$	$P2_12_12_1$	$P2_12_12_1$	$P2_12_12_1$
Unit cell / Å	a=120.5, b=227.9, c=286.8	a=122.2, b=228.5, c=286.7	a=121.2, b=228.2, c=287.0	a=123.3, b=229.1, c=286.8
Resolution / Å	50.0 - 2.15 (2.28 - 2.15)*	50 - 2.10 (2.18 - 2.10)*	50.0 - 2.15 (2.23 - 2.15)*	50.0 - 1.95 (2.02 - 1.95)*
No. of total reflections	2,282,747 (219,853)*	2,483,015 (237,353)*	2,926,414 (285,972)*	3,879,794 (364,987)*
No. of unique reflections	422,704 (41,787)*	460,342 (45,180)*	428,716 (42,587)*	583,452 (56,794)*
Redundancy	5.4 (5.3)*	5.4 (5.3)*	6.8 (6.7)*	6.6 (6.4)*
Completeness / %	99.3 (98.9)*	99.2 (98.1)*	99.9 (99.9)*	99.6 (97.7)*
R_{merge}	0.069 (1.470)*	0.072 (1.471)*	0.081 (1.472)*	0.078 (1.552)*
$CC_{1/2}$	0.999 (0.619)*	0.999 (0.653)*	0.999 (0.724)*	0.999 (0.681)*
Mean I/σ (I)	16.7 (1.5)*	14.2 (1.3)*	14.4 (1.5)*	14.8 (1.3)*
**Refinement statistics**				
Resolution / Å	50.0 - 2.15	50.0 - 2.10	50.0 - 2.15	50.0 - 1.95
R factor	0.149	0.157	0.151	0.155
R_{free}	0.185	0.191	0.185	0.185
No. of protein residues‡	5,276	5,274	5,210	5,276
No. of water molecules‡	3,082	2,573	2,454	3,088
Wilson B / $Å^2$	44.0	43.5	42.7	37.2
Average B (overall) / $Å^2$	57.1	59.3	64.8	51.9
Average B (protein) / $Å^2$	53.8	56.5	61.5	48.7
Average B (ligands) / $Å^2$	68.1	69.4	77.2	62.3
RMSD bond length / Å	0.008	0.008	0.009	0.009
RMSD bond angle / deg.	1.24	1.24	1.25	1.26
Ramachandran plot§				
Favored / %	98.0	98.3	98.2	98.2
Allowed / %	1.9	1.6	1.7	1.7
Outliers / %	0.1	0.1	0.1	0.1

	Control (Glycerol)	10 mM PPBQ (Glycerol) (PDB code: 8GN2)	1 mM PPBQ (Glycerol)
**Data collection statistics**			
Wavelength / Å	1.00	1.00	1.00
Space group	$P2_12_12_1$	$P2_12_12_1$	$P2_12_12_1$
Unit cell / Å	a=124.2, b=229.7, c=287.2	a=124.0, b=229.6, c=287.2	a=123.9, b=229.5, c=287.1
Resolution / Å	50.0 - 2.00 (2.07 - 2.00)*	50 - 1.95 (2.02 - 1.95)*	50.0 - 1.90 (1.97 - 1.90)*
No. of total reflections	3,753,232 (375,528)*	4,066,297 (403,326)*	4,384,064 (428,870)*
No. of unique reflections	546,900 (54,026)*	583,631 (57,320)*	628,623 (61,700)*
Redundancy	6.9 (6.9)*	7.0 (7.0)*	7.0 (6.9)*
Completeness / %	99.6 (99.1)*	98.7 (97.6)*	98.6 (97.4)*
R_{merge}	0.063 (1.068)*	0.061 (1.465)*	0.064 (1.445)*
$CC_{1/2}$	0.999 (0.827)*	1.000 (0.704)*	0.999 (0.706)*
mean I/σ (I)	18.1 (1.9)*	18.6 (1.4)*	17.8 (1.3)*
**Refinement statistics**			
Resolution / Å	50.0 - 2.00	50.0 - 1.95	50.0 - 1.90
R factor	0.152	0.153	0.151
R_{free}	0.183	0.181	0.179
No. of protein residues‡	5,273	5,272	5,275
No. of water molecules‡	2,938	3,237	3,168
Wilson B / $Å^2$	37.3	37.4	35.7
Average B (overall) / $Å^2$	53.2	53.1	50.3
Average B (protein) / $Å^2$	50.8	50.2	47.7
Average B (ligands) / $Å^2$	60.6	62.1	58.4
RMSD bond length / Å	0.008	0.008	0.008
RMSD bond angle / deg.	1.23	1.21	1.22
Ramachandran plot§			
Favored / %	98.3	98.3	98.1
Allowed / %	1.6	1.6	1.8
Outliers / %	0.1	0.1	0.1

B

	10mM DBBQ (anomalous)	10mM DCBQ (anomalous)
**Data collection statistics**		
Wavelength / Å	0.90	1.80
Space group	$P2_12_12_1$	$P2_12_12_1$
Unit cell / Å	a=122.2, b=228.5, c=286.7	a=122.4, b=228.5, c=286.6
Resolution / Å	50 - 2.15 (2.28 - 2.15)*	50 - 2.53 (2.60 - 2.53)*
No. of total reflections	3,622,704 (579,032)*	6,200,346 (414,656)*
No. of unique reflections	840,299 (133,543)*	607,435 (42,717)*
Redundancy	4.3 (4.3)*	10.2 (9.7)*
Completeness / %	97.6 (99.3)*	99.9 (100.0)*
R_{merge}	0.086 (1.128)*	0.222 (1.308)*
$CC_{1/2}$	0.998 (0.725)*	0.996 (0.822)*
mean I/σ (I)	10.99 (1.4)*	6.42 (1.6)*

*Values in the parentheses indicate those for the highest resolution shell.
‡Total number of residues of 2 PSII monomers in an asymmetric unit.
§Ramachandran plot was calculated with the MolProbility.

Table S2. The average B-factors ($Å^2$) of the Q_B and PPBQ molecules with different occupancy ratios.

**Occupancy ratio (PPBQ+Q_B)**	0+1.0	0.1+0.9	0.2+0.8	0.3+0.7	0.4+0.6	0.5+0.5	0.6+0.4
**1 mM PPBQ-treated**							
PPBQ	0	71.1	68.8	70.5	80.9	84.8	94.1
Q_B head	84.9	70.7	69.0	70.3	80.9	84.8	94.4
**10 mM PPBQ-treated**							
PPBQ	0	82.6	84.8	97.3	108.1	121.1	116.6
Q_B head	95.7	82.1	84.4	97.6	108.9	121,5	116.8
